# Retraction of “Long non-coding RNA TUG1 knockdown hinders the tumorigenesis of multiple myeloma by regulating microRNA-34a-5p/NOTCH1 signaling pathway”

**DOI:** 10.1515/biol-2021-0132

**Published:** 2021-12-21

**Authors:** Yongtian Zhang, Dandan Zhao, Shumei Li, Meng Xiao, Hongjing Zhou, Shuige Yang, Yunliang Hao, Shasha Dong

**Affiliations:** Department of Hematology, Ji’ning No. 1 People’s Hospital, Ji’ning, Shandong, China

**Retraction of:** Yongtian Zhang, Dandan Zhao, Shumei Li, Meng Xiao, Hongjing Zhou, Shuige Yang, Yunliang Hao and Shasha Dong. Long non-coding RNA TUG1 knockdown hinders the tumorigenesis of multiple myeloma by regulating microRNA-34a-5p/NOTCH1 signaling pathway. Open Life Sci. 15(1). doi: 10.1515/biol-2020-0025.

The authors and the publisher would like to inform that the “Long non-coding RNA TUG1 knockdown hinders the tumorigenesis of multiple myeloma by regulating microRNA-34a-5p/NOTCH1 signaling pathway” (doi: 10.1515/biol-2020-0025) has been retracted due to the authors admitting the scientific misconduct in this article. The authors apologize to the entire scientific community for any deception and misinformation caused by the article’s publication.

